# Performance-Based Functional Status Predicts Diffuse Cortical Atrophy in Alzheimer’s Disease

**DOI:** 10.3390/brainsci16030295

**Published:** 2026-03-06

**Authors:** Renata Kochhann, Patricia Ferreira da Silva, Eelco van Duinkerken, Maila Rossato Holz, Marcia Lorena Fagundes Chaves, Wyllians Vendramini Borelli, Rochele Paz Fonseca

**Affiliations:** 1 Psychology Department, Post-Graduate Program in Psychology, Human Cognition, Pontifícia Universidade Católica do Rio Grande do Sul (PUCRS), Porto Alegre 90619-900, Brazil; 2 Memory Center, Hospital Moinhos de Vento, Porto Alegre 90560-032, Brazil; 3 Department of Medical Psychology, Amsterdam University Medical Centers, Vrije Universiteit, 1081 HV Amsterdam, The Netherlands; 4 Post-Graduate Program in Neurology, Federal University of the State of Rio de Janeiro, Rio de Janeiro 21941-853, Brazil; 5 Hospital de Clínicas de Porto Alegre (HCPA), Porto Alegre 90035-903, Brazil; 6 Department of Morphological Sciences, Universidade Federal do Rio Grande do Sul (UFRGS), Porto Alegre 90010-150, Brazil; 7 Graduate Program in Biological Sciences: Pharmacology and Therapeutics (PPGFT), Universidade Federal do Rio Grande do Sul (UFRGS), Porto Alegre 90010-150, Brazil; 8 Medicine Department, Universidade Federal de Minas Gerais (UFMG), Belo Horizonte 31270-901, Brazil

**Keywords:** dementia, cognition, activities of daily living, brain cortical thickness

## Abstract

**Objectives:** We aimed to compare performance-based functional ability and cognitive screening performance to determine the cortical thickness relationship in cognitively unimpaired (CN) elders, mild cognitive impairment (MCI) and dementia patients, as well as to compare performance-based and proxy-evaluated functional ability and to determine its cerebral white and gray matter correlates. **Methods:** In total, 22 CN, 32 MCI, and 21 dementia patients were included in this study. They underwent clinical, cognitive, and Magnetic Resonance Imaging (MRI) assessment. Individuals were evaluated with the Mini-Mental State Examination (MMSE), the Rey Auditory Verbal Learning test (RAVLT), the Activities of Daily Living Questionnaire (ADL-Q) and the Direct Assessment of Functional Status-Revised (DAFS-R). **Results:** Higher ADL-Q scores were significantly associated with lower cortical thickness (bilateral temporoparietal regions, including the inferior temporal lobes and precuneus), *p* < 0.05. The DAFS-R scale showed a relationship with greater cortical thickness across extensive regions of the bilateral frontal, parietal, and temporal cortices (*p* < 0.05). MMSE presented a more focal association, primarily in canonical memory-related areas, including the medial and lateral temporal lobes and inferior parietal regions (*p* < 0.05). **Conclusions:** Functional independence measured by ADL-Q was associated with frontal and parietal cortical thickness, while DAFS-R scores demonstrated a more diffuse evaluation of cortical atrophy. Additionally, performance-based functional abilities according to the DAFS-R appear to be a stronger marker of cortical thickness than ADL-Q and MMSE.

## 1. Introduction

Cognition in dementia is traditionally evaluated by cognitive screeners tools such as Mini-Mental State Examination (MMSE). The Mini-Mental State Examination (MMSE) score has been positively correlated with cortical thickness, particularly in regions vulnerable to neurodegeneration such as the entorhinal cortex, hippocampus, parahippocampal gyrus, and other temporal and parietal areas. Lower MMSE scores are associated with reduced cortical thickness and gray matter volume in these regions, reflecting the anatomical substrate of global cognitive impairment seen in conditions like Alzheimer’s disease and mild cognitive impairment [1,2,3,4,5]. However, MMSE showed poor ability to discriminate against dementia and mild cognitive impairment (MCI) patients [6].

At the same time, functional ability is decisive in the clinical diagnosis of dementia and is traditionally evaluated by caregiver/proxy reporting. However, informant-reported measures of function may be limited by an unreliable reporter [7], causing informants to underestimate or overestimate the individual’s functional capacity [8]. Notwithstanding, with the increase in longevity and, consequently, the increase in elderly people living alone [9], assessment dependent on informants may not be the most accurate. However, just a few performance-based instruments have been validated and proven to be reliable in measuring Instrumental Activities of Daily Living (IADL) in Alzheimer’s Disease (AD) dementia [10]. IADLs are shown to be positively associated with cortical thickness in regions related to Alzheimer’s disease, such as the medial temporal lobes, cingulate cortex, and precuneus. Lower cortical thickness in these regions correlates with greater impairment in IADL performance, independent of age, sex, education, vascular injury, and intracranial volume [11]. This relationship is robust across the spectrum from subjective cognitive decline to mild cognitive impairment and Alzheimer’s dementia.

Cortical thickness and neuropsychological performance are key biomarkers for predicting the progression from MCI to dementia [12]. However, cognitive impairment in older adults with dementia has been studied more than functional assessments, where research remains relatively limited [12,13]. Some studies showed that reduced cortical thickness in an AD signature region and elevated brain amyloid were associated with faster functionality decline in participants without dementia at baseline [14]. Additionally, temporal atrophy was associated with IADL impairment in mild AD dementia at baseline, while baseline parietal and temporal atrophy, lower cerebrospinal fluid (CSF) Aβ1-42, and greater t-tau predict worsening IADL impairment over time across the AD spectrum [15]. Additionally, regional cortical thinning in temporal, parietal, frontal, and occipital areas—especially the fusiform gyrus—predicts variability in performance-based functional tasks, which are closely related to IADLs. These regions are implicated in executive, visuospatial, and learning functions that underpin complex daily activities [16]. In Alzheimer’s disease, the thinning of the dorsolateral prefrontal cortex and superior parietal cortex is linked to deficits in cognitive–motor automaticity and attention allocation, both of which are essential for successful IADL execution [17].

Although studies have been found that have assessed the association between cortical thickness and cognitive and functional performance, to the best of our knowledge, no study to date has established an association between performance-based assessment of functional performance and cortical thickness in older adults.

Therefore, we aimed to compare performance-based functional ability and MMSE performance to determine the cortical thickness relationship in cognitively unimpaired elders (CN) and MCI and dementia patients, as well as to compare performance-based and proxy-evaluated functional ability and to determine its cerebral white and gray matter correlates.

## 2. Materials and Methods

### 2.1. Participants and Procedures

Individuals attending a tertiary memory clinic (Hospital de Clínicas de Porto Alegre -HCPA) in Brazil were invited to participate consecutively from 2015 to 2016. They underwent a clinical, cognitive, and MRI assessment within a week. Community-dwelling controls were also invited to participate and recruited for this study. Diagnosis of dementia due to AD was based on the diagnostic criteria for Alzheimer’s disease [18]. MCI was defined according to the Petersen criteria [19]. Cognitively unimpaired (CN) elders were defined as individuals with the absence of uncorrected sensory disturbances (auditory and/or visual); the absence of current or previous neurological or psychiatric conditions that may interfere with their performance; the absence of a current or previous history of self-reported alcohol abuse, illicit drug use, and benzodiazepine use; the absence of depressive symptoms examined by the Beck Depression Inventory—BDI-II [20,21]; the absence of cognitive impairment assessed by the Mini-Mental State Examination [22,23,24]; and the absence of an intellectual level below the average examined by the Wechsler Abbreviated Scale of Intelligence (WASI) scale [25]. These elderly individuals were matched by age, education, and socioeconomic level in relation to the other groups. All participants and their family members/caregivers consented to participate in the research and provided written consent for this study. The study was approved by the Research Ethics Committee of the Pontifical Catholic University of Rio Grande do Sul (number 657.955, approved on 23 May 2014).

The authors used OpenEvidence (www.openevidence.com: accessed on 10 December 2025), an AI-powered tool that provides a platform for analyzing and organizing peer-reviewed medical literature, aiming to improve scientific writing during manuscript preparation. After using this tool, the authors carefully reviewed and edited the text and assume full responsibility for the final content.

### 2.2. Instruments

Individuals were evaluated with a cognitive battery that consisted of the following tests: Mini-Mental State Examination (MMSE) [22,23,24] as a cognitive screening; the Rey Auditory Verbal Learning test (RAVLT) [26,27] to measure immediate and delayed-recall memory scores; the Activities of Daily Living Questionnaire (ADL-Q) [28,29] as a gold standard for functional activity evaluation; and the Direct Assessment of Functional Status-Revised (DAFS-R) [30,31]. The DAFS-R is a performance-based instrument that evaluates the patient’s instrumental and basic activities of daily living based on the performance of activities that simulate everyday life. The domains assessed include temporal orientation, communication skills, finances, shopping skills, dressing skills, and eating skills. Functional impairment was evaluated by a rater team composed of neuropsychologists.

### 2.3. Structural MRI Acquisition and Analyses

All Magnetic Resonance Imaging (MRI) data were acquired at the Hospital de Clínicas de Porto Alegre (HCPA) on a 1.5T Philips Achieva scanner, equipped with a 32-channel head coil. High-resolution T1-weighted structural images were obtained for each participant using a volumetric Magnetization-Prepared Rapid Gradient-Echo (MPRAGE) sequence with the following parameters: Repetition Time (TR) = 8.6 ms; Echo Time (TE) = 3.99 ms; Inversion Time (TI) = 900 ms; flip angle = 8°; Field of View (FOV) = 256 × 256 mm; and acquisition matrix = 256 × 256. This protocol yielded 176 contiguous sagittal slices with a slice thickness of 1.0 mm, resulting in an isotropic voxel size of 1.0 × 1.0 × 1.0 mm^3^. All images were visually inspected for quality and motion artifacts prior to any subsequent analysis.

Structural T1-weighted images were processed using the FreeSurfer image analysis suite, version 7.2.0 (http://surfer.nmr.mgh.harvard.edu/) to generate cortical and subcortical segmentations. The automated processing pipeline included motion correction, non-brain tissue removal, intensity normalization, and segmentation of gray and white matter [32,33,34]. For our primary analysis, the cerebral cortex was parcellated into distinct regions of interest (ROIs) according to the Desikan–Killiany atlas [35]. The mean cortical thickness was extracted for each ROI, and the total hippocampal volume was extracted from the subcortical segmentation. Region of interest (ROI)-wise and vertex-wise analyses were conducted in this study due to their complementary approaches. While ROI analysis tests specific hypotheses with high sensitivity, vertex-wise analysis provide an unbiased evaluation of the whole cortex.

In addition to the ROI-based analysis, an exploratory vertex-wise analysis was conducted to identify localized, regional differences in cortical thickness across the entire cortical surface. A General Linear Model (GLM) was fitted at each vertex to assess group differences (CN vs. CI) in cortical thickness and regression model to identify predictors of cortical thickness (e.g., DAFS-R and ADL-Q as predictors). To account for multiple comparisons, a cluster-wise correction was applied using pre-computed Monte Carlo Null Z simulations. A cluster-forming threshold of *p* < 0.05 (−log10 = 1.3) was set, and clusters were considered significant at a cluster-wise probability (CWP) of *p* < 0.05, adjusted for both hemispheres (−2 spaces). All cortical reconstructions underwent rigorous visual inspection to ensure surface accuracy before statistical analysis (FreeSurfer v7.2.0).

### 2.4. Statistical Analysis

A descriptive analysis was performed to characterize the study sample. Continuous variables, including age and scores from the cognitive testing are presented as mean ± standard deviation (M ± SD). Categorical variables, such as sex was reported as frequencies and percentages (n, %). Pearson’s correlation coefficient was calculated between demographic (age, education), cognitive (MMSE, RAVLT-A7 list), and functional (DAFS, ADL-Q) scores.

To identify significant predictors of regional brain structure (gray cerebral cortical thickness, total cerebral cortical thickness and bilateral hippocampal cortical thickness), a series of simple linear regression analyses (enter method) were conducted. For each analysis, the cortical thickness of a specific region of interest (ROI) was entered as the dependent variable including age, education, ADL-Q, DAFS-R and MMSE as a predictor for each analysis. All statistical analyses were conducted using Statistical Package for the Social Sciences (SPSS) version 23.0 (IBM Corp., Armonk, NY, USA). The significance level for all statistical tests was set at an alpha of α = 0.05.

## 3. Results

The final study sample comprised 75 participants, categorized into three diagnostic groups: 22 presenting CN, 32 presenting MCI, and 21 presenting dementia. The CN and MCI groups were comparable in age (66.0 ± 0.5 and 66.5 ± 0.5 years, *p* > 0.05), while the dementia group was older (75.0 ± 0.4 years). The overall sample was predominantly female (69.3%). As expected, a clear gradient was observed across the clinical spectrum for years of education, cognitive performance (MMSE and RAVLT delayed recall), and functional scores (DAFS-R and ADL-Q, Table 1).

The correlation between demographic (age, education), cognitive (MMSE, RAVLT-A7 list), and functional (DAFS, ADL-Q) scores is presented in Supplementary Figure S1.

Simple linear regression analysis was conducted for each variable. The variance in the total cerebral cortical thickness was explained approximately 54% by age (F(1,73) = 60.72, *p* < 0.001), 14% by education (F(1,73) = 11.74, *p* = 0.001), 27% by MMSE (F(1,72) = 26.08, *p* < 0.001), 22% by ADL-Q (F(1,68) = 19.60, *p* < 0.001), 42% by DAFS-R (F(1,52) = 38.06, *p* < 0.001). The variance in the gray cerebral cortical thickness was explained approximately 40% by age (F(1,73) = 48.45, *p* < 0.001), 10% by education (F(1,73) = 8.56, *p* = 0.005), 26% by MMSE (F(1,72) = 25.45, *p* < 0.001), 28% by ADL-Q (F(1,68) = 26.20, *p* < 0.001), 50% by DAFS-R (F(1,52) = 52.71, *p* < 0.001). The variance in the bilateral hippocampal cortical thickness was explained approximately 20% by age (F(1,73) = 18.44, *p* < 0.001), 15% by MMSE (F(1,72) = 12.68, *p* = 0.001), 27% by ADL-Q (F(1,68) = 24.93, *p* < 0.001), 28% by DAFS-R (F(1,52) = 20.15, *p* < 0.001), and not by education (F(1,73) = 1.89, *p* = 0.173). DAFS-R presented an important role in predicting each cerebral cortical thickness area, as well as age, except in the hippocampus area. On the other hand, education presented the least variance for these cortical areas; more information is presented in Table 2.

**Figure 1:**
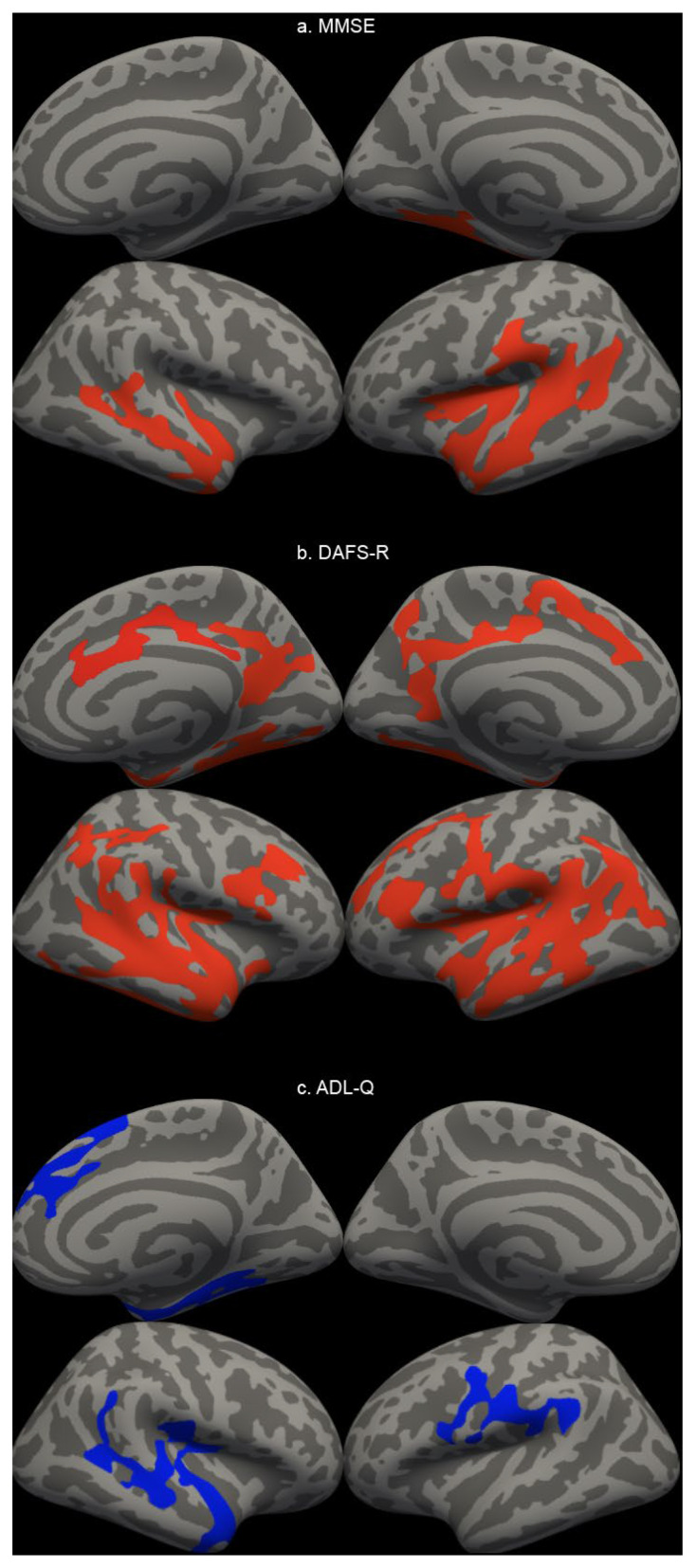
Surface-based morphometry analysis of clinical and functional scales. Note: General Linear Model maps displaying the clusters of association between cortical thickness and (**a**) Mini-Mental State Examination (MMSE), (**b**) DAFS-R total score, and (**c**) Activities of Daily Living Questionnaire (ADL-Q) bilaterally. Results are overlaid on the brain inflated surface. Red indicates a significant positive correlation, while blue indicates a significant negative correlation. Abbreviations: LH, left hemisphere; DAFS-R, Direct Assessment of Functional Status-Revised; ADL-Q, Activities of Daily Living Questionnaire; MMSE, Mini-Mental State Examination.

The whole-brain vertex-wise analyses revealed distinct patterns of association between cortical thickness and two functional measures (ADL-Q and DAFS-R) and a cognitive screening (MMSE), as illustrated in Figure 1. As shown, higher ADL-Q scores (indicating worse daily functioning) were significantly associated with lower cortical thickness (*p* < 0.05 corrected). These associations were anatomically specific, primarily localized to bilateral temporoparietal regions, including the inferior temporal lobes and precuneus. In contrast, the analysis with the DAFS-R scale revealed a much more widespread relationship. Higher DAFS-R scores (reflecting better functional ability) were positively associated with greater cortical thickness across extensive regions of the bilateral frontal, parietal, and temporal cortices (*p* < 0.05 corrected). In contrast, the association for the MMSE was significantly more focal. Higher MMSE scores were linked to greater thickness primarily in canonical memory-related areas, including the medial and lateral temporal lobes and inferior parietal regions (*p* < 0.05 corrected). The whole-brain vertex-wise cortical thickness comparisons across diagnostic groups is presented in Supplementary Figure S2. And the summary of significant clusters of cortical thickness analyzed is presented in Supplementary Table S1.

## 4. Discussion

To the best of our knowledge, this is the first study that provided structural data associated with performance-based functional ability measured by DAFS-R. Our findings showed that DAFS-R scores were significantly associated with a more diffuse cortical thickness than MMSE and ADL-Q were. Functional independence measured by ADL-Q was associated with frontal and parietal cortical thickness, while DAFS-R scores demonstrated a more diffuse evaluation of cortical atrophy across extensive regions of the bilateral frontal, parietal, and temporal cortices. Thus, performance-based evaluation of functional abilities by the DAFS-R appears to be a stronger marker of cortical thickness than ADL-Q (proxy reporting).

The DAFS-R is a scale that assesses both basic and instrumental activities, similar to the ADL-Q. Both scales have the same aim, though they present differences regarding scores, such as the inverted scoring system and different information sources. The DAFS-R showed a stepwise decrease across disease severity, whereas the Activities of Daily Living Questionnaire (ADL-Q) primarily identified individuals with dementia. The DAFS-R, as a performance-based measure, is sensitive to gradations in functional impairment and can differentiate between normal aging, MCI, and dementia, showing a progressive decline in scores as disease severity increases [8,31]. In contrast, the ADL-Q, which is informant-based, demonstrates high validity and reliability for identifying functional impairment in dementia but is less sensitive to early or subtle changes, thus primarily distinguishing individuals with established dementia from those without [28,36]. However, to date, the neurobiological correlation with direct functional independence measured by DAFS-R remains unclear.

In this sample, DAFS-R scores were significantly associated with diffuse cortical thickness in both vertex-wise and ROI-wise analyses. Moreover, DAFS-R scores predicted brain structure in a wider and more comprehensive manner than the ADL-Q scores, indicating that performance-based functional measures might be more accurate in identifying AD-related brain changes. Similarly, DAFS-R scores showed a broader picture of brain changes associated with AD atrophy than the MMSE association in both statistical analyses. Previous studies have demonstrated that MMSE scores were an important predictor of cortical thinning in AD. Yamashita et al. (2022) found that MMSE was correlated with the right entorhinal cortical thickness, while Yao et al. (2012) reported a relation with the left superior and left middle temporal gyrus, and Apostolova et al. (2006) presented an association with the entorhinal, parahippocampal, precuneus, superior parietal, and subgenual cingulate/orbitofrontal cortices [1,2,3]. Our findings present a similar relationship between MMSE and cortical thickness, including the medial and lateral temporal lobes and inferior parietal regions.

Brain areas associated with DAFS-R demonstrated a strong association with AD-related brain and clinical changes. Regional atrophy was associated with worse DAFS-R scores, including both lateral temporal lobes, medial temporal regions (hippocampus, parahippocampus and entorhinal gyri), precuneus, and posterior cingulate. It is well established that most characteristic regions of brain atrophy in AD are the hippocampus, entorhinal cortex, parietal cortex (especially the precuneus and posterior cingulate), and lateral temporal cortex [13,37,38]. Our findings corroborate the same pattern of atrophy demonstrated in these studies, suggesting that DAFS-R can be a strong predictor of AD-related brain atrophy.

This study also presents limitations. DAFS-R is helpful in staging dementia severity but may not be sensitive enough to detect very early ADL impairment at the stage of MCI [10]. However, it is the only scale validated in Brazil, and therefore a new scale is in the validation process to remedy this weakness [39]. Moreover, since AD diagnostic criteria change over time, the study’s participants may not match the latest guidelines [40]. Additionally, other factors should also be acknowledged in this study: the cross-sectional design, absence of biomarker validation (amyloid or tau PET), and limited generalizability beyond the Brazilian population. As well, age differences should also be considered when evaluating cortical thickness, despite its inclusion as a covariate. Furthermore, education should also be investigated more thoroughly due to its important role in relation to cognitive reserve [41].

## 5. Conclusions

In summary, performance-based functional ability with DAFS-R identified a diffuse cortical thickness and may suggest early spread-out atrophy, suggesting that DAFS-R has potential in identifying brain changes associated with early functional impairment. Further studies should evaluate earlier AD stages with biomarker confirmation of amyloid and tau pathology, as well as use in different dementia staging.

## Figures and Tables

**Table 1:** Sample demographics.

	CN (n = 22)	MCI (n = 32)	Dementia (n = 21)
Age, mean years ±SD	66 ± 0.5	66.5 ± 0.5	75 ± 0.4
Female, n (%)	14 (63.6)	22 (68.7)	16 (76.2)
Years of education, mean ±SD	15 ± 5.3	11.5 ± 5.3	5 ± 6.5
MMSE scores, mean ± SD	29 ± 1.3	26.5 ± 3.4	21 ± 4.4
RAVLT delayed recall, mean ± SD	9 ± 2.1	5 ± 2.9	0 ± 2.4
DAFS-R total score, mean ± SD	93.1 ± 5.1	82.8 ± 12.4	57.0 ± 13.2
ADL-Q, mean ± SD	2.35 ± 3.5	7.91 ± 8.1	42.18 ± 12.2

Note: CN = cognitively unimpaired elders, MCI = mild cognitive impairment, MMSE = Mini-Mental State Examination, DAFS-R = Directed Assessment Functional Status revised, ADL-Q = Activities of Daily Living Questionnaire.

**Table 2:** Predictors of cortical thickness through linear regression analysis.

Dependent Variable	Predictors	B	SE	95% CI		*ß*	*p*
				LL	UL		
Total cerebral cortical thickness	Age	−7470.10	958.64	−9380.67	−5559.53	−0.67	<0.001
	Education	4577.27	1335.92	1914.78	7239.76	0.37	0.001
	MMSE	9210.59	1803.41	5615.55	12,805.64	0.52	<0.001
	ADL-Q	−1974.27	445.91	−2864.07	−1084.48	−0.47	<0.001
	DAFS-R	2839.07	460.20	1915.62	3762.53	0.65	<0.001
Gray cerebral cortical thickness	Age	−5486.80	788.27	−7057.81	−3915.79	−0.63	<0.001
	Education	3122.28	1067.08	995.59	5248.96	0.32	0.005
	MMSE	7148.18	1416.84	4323.76	9972.61	0.51	<0.001
	ADL-Q	−1703.81	332.85	−2368.01	−1039.61	−0.53	<0.001
	DAFS-R	2350.93	323.82	1701.13	3000.74	0.71	<0.001
Bilateral hippocampal cortical thickness	Age	−48.13	11.21	−70.46	−25.79	−0.45	<0.001
	Education	18.90	13.74	−8.48	46.28	0.16	0.173
	MMSE	66.79	18.75	29.40	104.18	0.39	0.001
	ADL-Q	−20.51	4.11	−28.71	−12.32	−0.52	<0.001
	DAFS-R	21.02	4.68	11.62	30.41	0.53	<0.001

Note. ADL-Q = Activities of Daily Living Questionnaire, DAFS-R = Directed Assessment Functional Status revised.

## Data Availability

The data presented in this study are available on request from the corresponding author due to containing information that could compromise the privacy of research participants.

## Supplementary Materials

The following supporting information can be downloaded at: https://www.mdpi.com/article/10.3390/brainsci16030295/s1, Figure S1: Correlation matrix between demographic (age, education), cognitive (MMSE, RAVLT-A7 list), and functional (DAFS, ADL-Q) scores. Pearson’s correlation coefficient was calculated. Figure S2. Whole-brain vertex-wise cortical thickness comparisons across diagnostic groups. Table S1. Summary of significant clusters of cortical thickness analysed in the group comparison analyses, with mean effect sizes found.
