# Supplementary material for: Performance-Based Functional Status Predicts Diffuse Cortical Atrophy in Alzheimer’s Disease
Source: Brain Sci. 2026 Mar 6;16(3):295. doi: 10.3390/brainsci16030295 (PMC13024149; doi:10.3390/brainsci16030295)
Supplement: Supplementary file 1 [file brainsci-16-00295-s001.zip › brainsci-4081311-supplementary.pdf]

Title: Performance-based Functional Status Predicts diffuse cortical atrophy in Alzheimer's Disease

Authors
Renata Kochhann[1,2*], Patricia Ferreira da Silva[1], Eelco Van Duinkerken[3,4], Maila Rossato Holz[1], Marcia Lorena Fagundes Chaves5, Wyllians Vendramini Borelli[2,6,7#], Rochele Paz Fonseca[1,8#]

Affiliations:
[1] Pontifícia Universidade Católica do Rio Grande do Sul (PUCRS), Porto Alegre, Brazil

[2] Memory Center, Hospital Moinhos de Vento, Porto Alegre, RS, Brazil.

[3] Department of Medical Psychology, Amsterdam University Medical Centers, Location Vrije Universiteit, Amsterdam, the Netherlands

[4] Post-graduate program in Neurology, Federal University of the State of Rio de Janeiro, Rio de Janeiro, Brazil

[5] Hospital de Clínicas de Porto Alegre (HCPA), Porto Alegre, Brazil

[6] Department of Morphological Sciences, Universidade Federal do Rio Grande do Sul (UFRGS), Porto Alegre, RS, Brazil.

[7]Graduate Program in Biological Sciences: Pharmacology and Therapeutics (PPGFT), UFRGS, Porto Alegre, RS, Brazil.

[8] Universidade Federal de Minas Gerais (UFMG), Belo Horizonte, Brazil

# These authors have contributed equally as last authors.

Supplementary Figure S1. Correlation matrix between demographic (age, education), cognitive (MMSE, RAVLT-A7 list), and functional (DAFS, ADL-Q) scores. Pearson's correlation coefficient was calculated.

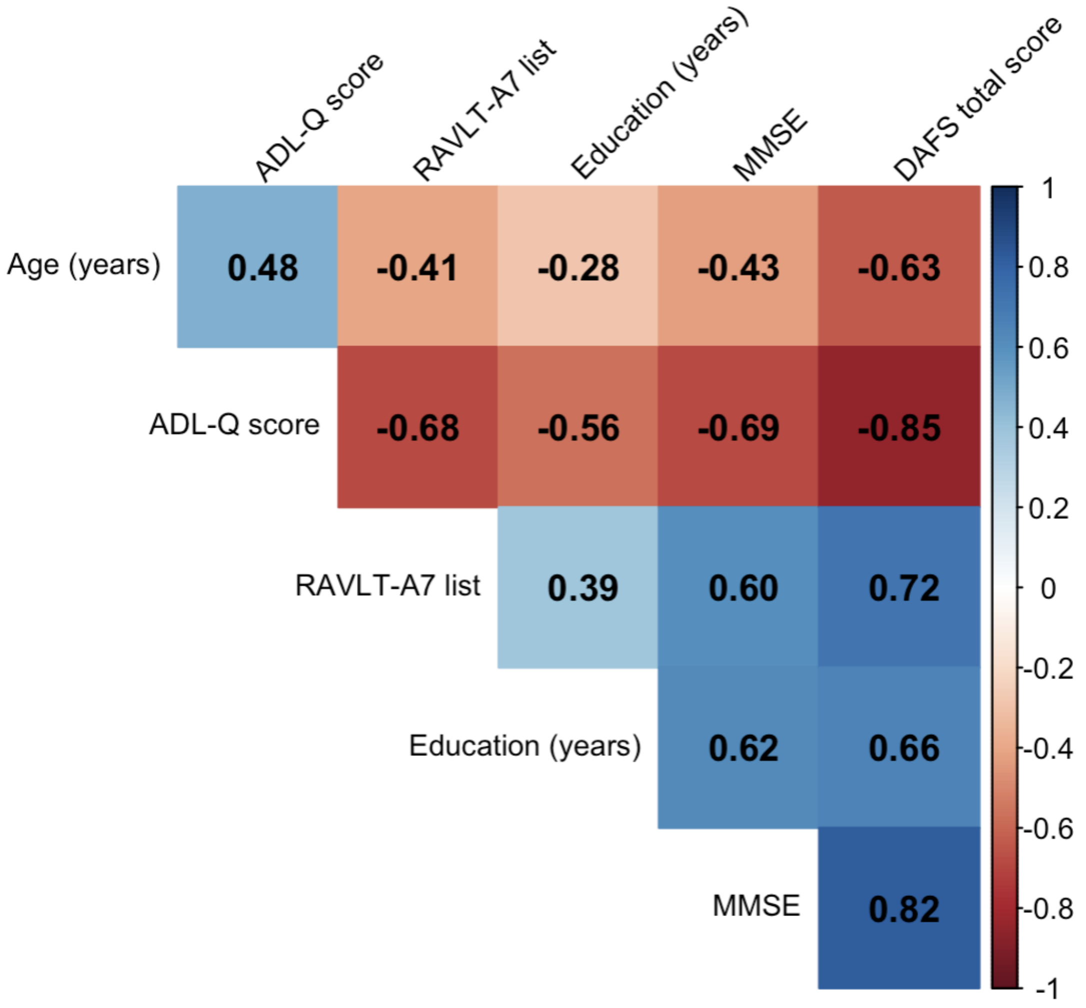

Note: MMSE= Mini-mental State Examination, DAFS= Directed Assessment Functional Status revised, ADL-Q= Activities of Daily Living Questionnaire, RAVLT-A7= Rey Auditory-Verbal Learning Test - A7 list.

Supplementary Figure S2. Whole-brain vertex-wise cortical thickness comparisons across diagnostic groups.

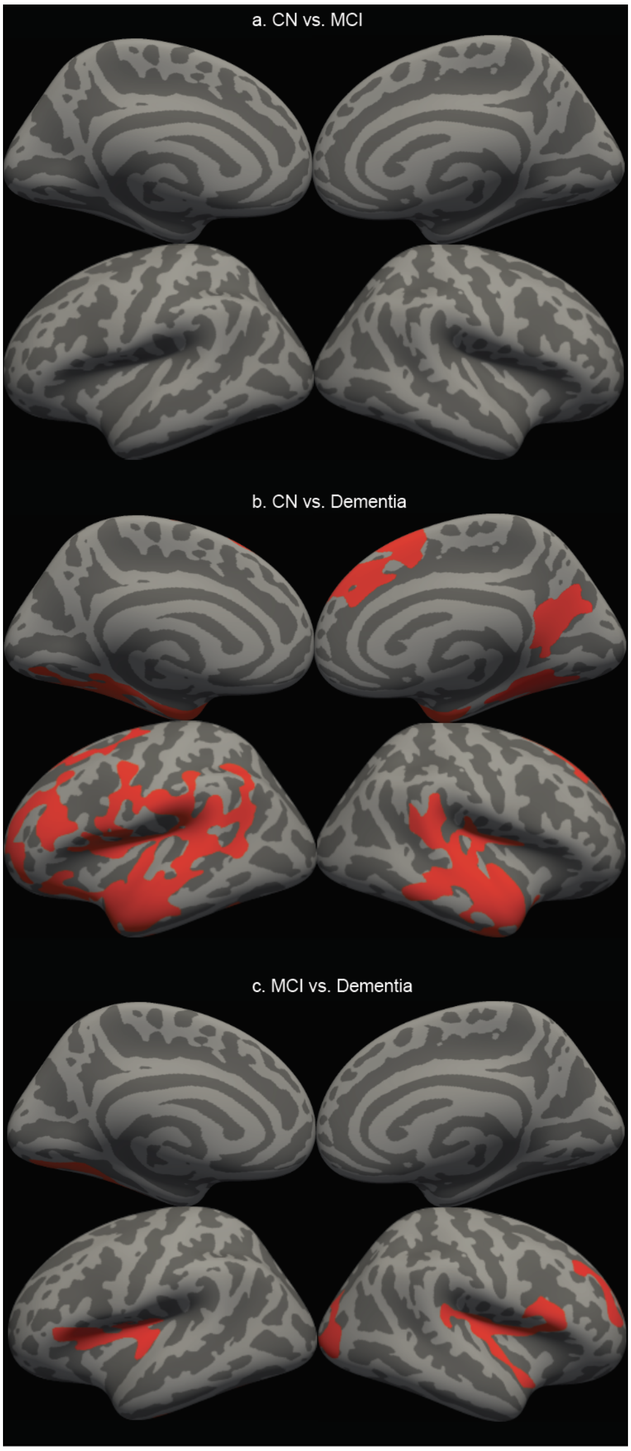

Note: Surface maps showing significant differences in cortical thickness between (a) Cognitively Normal (CN) vs. Mild Cognitive Impairment (MCI), (b) CN vs. Dementia, and (c) MCI vs. Dementia. Statistical maps are displayed on the brain inflated surface, showing lateral and medial views for both hemispheres. The red color represents areas of significantly reduced cortical thickness in the more advanced diagnostic group of each pair (e.g., areas where Dementia < CN).

Supplementary Table S1. Summary of significant clusters of cortical thickness analysed in the group comparison analyses, with mean effect sizes found.

	MNI coordinates					
**Cluster**	**X**	**Y**	**Z**	**Region**	**Mean effect size**	**P-values**
**DAFS-R**						
Left 1	6.9964	30124	68519.65	insula	0.04	<0.001
Left 2	3.9716	7106	14498.52	posteriorcingulate	0.12	<0.001
Left 3	6.0451	3564	8425.38	fusiform	0.42	<0.001
Right 1	7.5703	25427	60963.61	postcentral	0.05	<0.001
Right 2	4.0561	4367	9176.45	supramarginal	0.44	<0.001
Right 3	5.5647	3502	8341.48	precuneus	0.6	<0.001
Right 4	4.2861	3199	6808.19	parsopercularis	0.07	<0.001
Right 5	2.8446	2919	5272.39	caudalanteriorcingulate	0.09	<0.001
Right 6	2.8885	1921	3571.77	superiorfrontal	0.24	<0.001
**ADL-Q**						
Left 1	-43.481	5080	-9981.81	supramarginal	0.46	<0.001
Left 2	-31.555	2232	-4008.10	superiorfrontal	0.04	<0.001
Right 1	-48.586	10035	-20537.24	superiortemporal	0.16	<0.001
Right 2	-46.813	4121	-9095.86	entorhinal	0.06	<0.001
Right 3	-38.766	3533	-6591.18	superiorfrontal	0.35	<0.001
**MMSE**						
Left 1	5.5236	17250	40558.59	middletemporal	0.12	<0.001
Left 2	7.0915	4010	9406.10	fusiform	0.43	<0.001
Right 1	4.6836	7532	15871.70	inferiortemporal	0.03	<0.001
